# Difficulties in maintaining electroconvulsive treatment (ECT) in a psychiatric hospital during covid19 pandemic

**DOI:** 10.1192/j.eurpsy.2021.707

**Published:** 2021-08-13

**Authors:** M. Rojo, M. Constantin, B. Langree, N. Marie, R. Bellay

**Affiliations:** 1 Pharmacie, centre hospitalier Guillaume Régnier, rennes, France; 2 Pharmacie, Centre hospitalier Guillaume Regnier, Rennes, France

**Keywords:** ECT, Pharmacy, COVID-19, supply

## Abstract

**Introduction:**

ECT is an effective care with high level of recommendation. During the COVID19, new recommendations to protect patients and caregivers combined with the increasing use of medicines and medical devices (MD) for anesthesia, caused greater difficulties of supply. Even if vital for patients, it is challenging to maintain ECT in this environment.

**Objectives:**

The aim of this study is to resume the measures implemented in order to maintain ECT during COVID19.

**Methods:**

Retrospective analysis of measures implemented to maintain the ECT during COVID19.

**Results:**

As FFP2 masks were restricted to intensive care units, our hospital were not supplied. After negotiations, the regional health agency (ARS) has granted us an allocation of 100 masks to maintain ECT. Our efficient stock management of personal protective equipment as well as our transparency on these stocks with ARS and sharing with other hospitals out of stock played a role in this agreement.We had to adapt our MDs references according to breaks of many ones and new recommendations. The university hospital helping us in supplying certain missing references. Considering the difficulties in supplying drugs and MDs, and limited availability of anesthetists, we have reduced the number of ECT. Prioritization of patients with vital indications had to be achieved.

**Conclusions:**

The prioritization of some services by the regulatory agency causes many supply difficulties for the others. It would be important to reassess the priority of ECT in such crisis because most of the time other caregivers and regulatory agencies are not aware how they are vital for patients.

